# Rapid emergence of cryptococcal fungemia, Mycobacterium chelonae vertebral osteomyelitis and gastro intestinal stromal tumor in a young HIV late presenter: a case report

**DOI:** 10.1186/s12879-018-3573-z

**Published:** 2018-12-27

**Authors:** Andrea Marino, Eleonora Caltabiano, Aldo Zagami, Anna Onorante, Carmela Zappalà, Maria Elena Locatelli, Alessio Pampaloni, Daniele Scuderi, Roberto Bruno, Bruno Cacopardo

**Affiliations:** Division of Infectious Diseases, Department of Clinical and Experimental Medicine, ARNAS Garibaldi Hospital, University of Catania, Catania, Italy

**Keywords:** HIV, Late presenters, Opportunistic infections, Cryptococcosis, Non-tuberculous mycobacteria, GIST

## Abstract

**Background:**

Highly active antiretroviral therapy has significantly changed the natural history of HIV infection, leading to a dramatic reduction of HIV-related morbidity and mortality. Late Presenters, Very Late Presenters and AIDS presenters still represent, also in Europe, including Italy, a huge challenge in terms of diagnostic and therapeutic management.

**Case presentation:**

A 35-year-old male with a history of fever and back pain. HIV test resulted positive with a high HIV Viral Load and a very low T-CD4 number of cells (5 cells/mm^3^). Imaging investigations revealed multiple vertebral and pulmonary lesions together with abdominal and thoracic lymphadenopathy. Blood cultures were positive for *Cryptococcus neoformans* and for *Staphylococcus haemolyticus*.

Lymphnode biopsy resulted positive in PCR for Non-Tuberculosis Mycobacteria (*Mycobacterium chelonae*). A gastric biopsy also revealed a GIST. The patient also had CMV DNA positive. Although we performed antiretroviral therapy and specific-therapies for each disease, he was transferred to intensive care unit where he died due to an Acute Respiratory Distress Syndrome.

**Conclusion:**

The reported case is unusual due to the relevant number of opportunistic diseases (both infectious and tumoral) emerging not long after the HIV infection had been diagnosed.

Late presenters HIV patients and AIDS presenters still represent a challenge, which is often too complex for clinicians to deal with. In spite of proper management, the risk of suboptimal results cannot be excluded.

**Electronic supplementary material:**

The online version of this article (10.1186/s12879-018-3573-z) contains supplementary material, which is available to authorized users.

## Background

Highly active antiretroviral therapy (HAART) has significantly changed the natural history of HIV infection, leading to a dramatic reduction of HIV-related morbidity and mortality [1, 2] however, even today, Late Presenters (LP) and AIDS presenters still represent a huge challenge for clinicians.

According to the definition approved by the European Consensus panel, Late Presenters are defined as patients, who are in need of care, with a CD4+ T-cell count below 350 cells/μl or with an AIDS-defining event regardless of the CD4+ T-cell count. Very late presenters (VLP) or patients with advanced HIV disease (AHD) are those with a CD4+ T-cell count below 200 cells/μl or AIDS, regardless of CD4+ T-cell count [1, 3].

Failure to reach an early HIV diagnosis is associated with increased morbidity and mortality [4] as well as with increased healthcare costs [5, 6] and a relevant risk of onward transmission [7].

A number of short term implications due to late presentation should be considered, such as an increased risk of mortality throughout the first year, an increased risk of HIV-related clinical events in the first 3 months, a greater number of hospitalizations in the first year, a reduced chance of viral suppression on HAART, blunted/delayed CD4 responses on HAART and an increased potential for drug–drug interactions and overlapping toxicities [8].

On the other hand, long term implications are represented by a higher risk of mortality over a longer period (3 years), possible increased risk of some non-AIDS events, increased risk of cognitive impairment and increased hospital care/drug costs over 7–8 years. [8].

Previous cohort studies showed that the risk of death after HAART initiation was higher in subjects starting therapy with CD4 + T-cell counts below 350/mm^3^ as compared to subjects starting therapy with CD4 + T-cell counts above this level [9, 10].

About half (51%) of those diagnosed with HIV in 2016 in the European Region were LP [11]. The percentage of late presentation in Italy in 2016 was 55.6% while 36.9% were VLP [12]. In 2016, in Europe, 3628 individuals were diagnosed as being affected with AIDS [11], 778 of these case of AIDS were diagnosed in Italy [12].

Here we report a case of a HIV Late Presenter patient with a number of severe and uncommon comorbidities that, despite receiving the appropriate therapies, led to his rapid death.

## Case presentation

On December 2017, a 35-year old male was admitted into a peripheral hospital in northern Sicily, the patient had been suffering from a fever with chills (peaking to 39 °C) and back pain in the lumbar and dorsal area, for as long as 7 days.

He had an unremarkable clinical history, except for minor surgery because of anal fissure in November 2017.

After admission, the patient started, unsuccessfully, oral levofloxacin 500 mg/day and ceftriaxone 1 g/day intramuscularly.

A MRI of the vertebral column was performed showing vertebral lesions affecting multiple vertebral bodies (from D8 to L5). The major lesions were target-like and showed a peripheral hypointense circle in T2 sequence.

Due to the finding of leukopenia with lymphopenia, HIV serology was performed resulting positive. Viral Load (VL) was 4,370,000 copies/ml and T CD4+ count was 5 cells/μL. Antiretroviral therapy with emtricitabine/tenofovir disoproxil, ritonavir and darunavir was started.

On January 11th, with the hypothesis of bacterial spondylitis, the patient started an empirical antibiotic therapy with rifampicin and teicoplanin.

On January 17th, due to the persistence of fever and back pain, the patient was transferred to the Infectious Diseases Unit of Garibaldi Hospital in Catania (eastern Sicily).

On admission the patient was febrile (T. max 38,3 °C) with intense pain in the dorsal and lumbar area; blood pressure was 95/40 mmHg, heart rate was 124 bpm. Oxygen saturation was 97% in room air. Neurocognitive function was preserved as assessed by Mini Mental State Examination (MMSE) and International HIV-Dementia Scale (IHDS).

He vigorously and repeatedly denied any risk factor for exposure to HIV infection. He had no previous history of drug abuse, he denied having any type of sexual relationships with both females and males and he had never used blood products. He had not tattoos or piercings. He denied smoking or drinking alcohol. He was allergic only to strawberries.

Moreover, he had never had major surgery and he had never undergone any medical invasive procedures. He had no comorbidities so he was not taking any drugs or medication.

His medical family history was unremarkable.

Laboratory examinations showed normocytic anemia (Hb: 6,7 g/dl, RBC: 2,45 * 10^6^/mm^3^), leukopenia with neutropenia (WBC: 1700/mm^3^), low platelet count (106,000/mm^3^); high values of CRP, ESR and ferritin levels.

Hepatitis B and Hepatitis C virus serology was negative, as well as TPHA and VDRL. Serum 1–3 Beta D glucan was < 60 pg/mL and Galactomannan was 0.08 Optical Density Index.

ART was immediately switched to emtricitabine/tenofovir alefanamide plus dolutegravir, in order to reduce pharmacological impact on bones and to preserve kidney function.

On January 19th, the patient performed a total body CT scan which showed multiple bilateral lung nodular lesions with paratracheal and mediastinal lymphadenomegaly (around 1 cm diameter); presence of multiple hypodense lesions involving spleen and liver (diameter between 1.5 and 2 cm). Mesenteric, iliac and lombo-aortic lymphadenomegaly was also present with a diameter of > 1 cm.

On January 20th, eight out of eight blood cultures gave a positive result for *Cryptococcus neoformans*; thus, induction therapy with liposomal amphotericin 3 mg/kg/die plus fluconazole 800 mg intravenously was started on January 21st obtaining a prompt remission of fever and rapid disappearance of lung nodular lesions. The induction therapy was administered for as long as 15 days. A lumbar puncture was performed but none of the cultures, antigenic tests nor the India Ink stain on CSF resulted negative for *Cryptococcus.* After the induction therapy, a series of repeated blood cultures resulted negative for *Cryptococcus* so maintenance therapy was started with fluconazole 400 mg.

On January 30th, due to a recurrence of fever and chills, additional blood cultures were performed resulting positive for *Staphylococcus haemolyticus*: daily doses of levofloxacin (750 mg) and teicoplanin (400 mg) were started.

Fever disappeared again within 3 days of antibiotics administration.

On February 5th, a vertebral biopsy on the affected bodies (L2-L3 tract) was performed as well as a bone marrow biopsy: in both tissues, both Real Time PCR and cultures gave a positive result for Non-Tuberculosis Mycobacteria (NTM) (in detail, *Mycobacterium chelonae*).

On February 19th, following Mycobacteria isolation, intravenous tobramicin 80 mg tid was administered together with oral clarithromycin (500 mg twice daily). Within ten days from the onset of therapy, the patient claimed to have had a significant relief from dorsal and lumbar pain.

Meanwhile (February 28th), due to the occurrence of epigastric pain with retrosternal pirosis, an oesophagogastroduodenoscopy was performed which showed a single ulcerated lesion 1.3 cm long in the great curvature of the stomach. Ulcer biopsy showed epithelioid and spindle-shaped cells with nuclear atypia and the immuno-phenotypic profile was positive for a GIST pattern: CD117, CD34, Ki67 10%.

In spite of targeted multidrug treatment, fever appeared again (over 38 °C) and on March 24th, the patient revealed acute dyspnea with blood gas analysis showing low PaO_2_ (60.3 mmHg) and low PaCO2 (27.4 mmHg), blood pH was 7.41. An urgent chest X-Ray showed bilateral lung reticolo-nodular infiltrates with the characteristics of an acute respiratory distress syndrome (ARDS).

Real Time PCR and O-toluidine smears for Pneumocystis Jirovecii on sputum resulted negative whereas CMV DNA was detected as being positive with 1094 UI/mL.

The patient was transferred to the intensive care unit (ICU) on the same day but, in spite of assisted ventilation and life supports, he died within 5 days of his ICU admission (Additional files 1 and 2).

## Discussion and Conclusions

HIV Late Presenters represent a population at major risk for severe and multiple Opportunistic Infections (OIs) and tumors occurrence.

Cryptococcosis represents one of the most severe OIs whose diagnostic and therapeutic management remains challenging. Without appropriate treatment, cryptococcosis in HIV infected patients has a very high mortality rate [13, 14]. Usually, *Cryptococcus* causes focal infections in AIDS patients such as meningoencephalitis or pneumonia or nodular skin lesions [15–18]. In the case presented here, a disseminated cryptococcal infection occurred with overt cryptococcemia and probable coexisting pulmonary lesions. Rigby et al. described a rare case of cryptococcemia with contemporary lung involvement in a HIV young male patient presenting with a miliary-like pattern [19].

Prompt management of the disease might be essential to improve the patients’ survival and quality of life [20].

Nontuberculous mycobacteria (NTM) are also important causes of pulmonary and extra pulmonary diseases in immunosuppressed hosts [21]. *Mycobacterium chelonae* is ubiquitous in the environment and it is commonly associated with skin and soft tissue infections. Although rarely seen, invasive infections may include osteomyelitis with particular reference to HIV-infected individuals with severe immunosuppression [22–24]. Gadre et al. reported a case of a 32-year-old HIV positive male who presented an atypical osteomyelitis of the-right tibia due to *M. fortuitum-chelonae* group infection [23]. Also, Rahman et al. reported a case of *M. chelonae* vertebral osteomyelitis in a man with a previous history of intravenous drug abuse [24]. Another case was reported by Korres et al. who used clarithromycin (500 mg twice daily) and amikacin (1 g daily) to treat a severe spondylodiscitis by *Mycobacterium chelonae* in a patient undergoing immunosuppressive therapy following renal transplantation [25].

Actually, in our case, soon after the start of antimycobacterial treatment, a relevant decrease of back pain was noticed. Isolates of M. chelonae are usually susceptible to tobramycin (100%), clarithromycin (100%), linezolid (90%), imipenem (60%), amikacin (50%), clofazimine, doxycycline (25%), and ciprofloxacin (20%) [26].

Though the introduction of highly active antiretroviral therapy (HAART) diminished the importance of NTM in the setting of human immunodeficiency virus (HIV), the management of the wide spectrum of diseases caused by non-tuberculous mycobacteria is still challenging due to the extended antibiotic-resistance as well as the poor clinical conditions of the patient.

Gastrointestinal stromal tumors (GISTs) are the most common mesenchymal tumors of the gastrointestinal tract, 60% involving the stomach and 30% in the small bowel; 25% of GISTs are asymptomatic and accidentally discovered, 10–20% of them reveal metastatic disease upon initial diagnosis [27]. As far as we know, only few cases of GIST in HIV positive patients have been reported. Padula et al. described a rare case of malignant GIST in the esophagus of a HIV positive male patient that resulted in an extremely unusual metastatic site [28]. Also Kubben et al. reported the case of a young AIDS patient presenting with GIST in the small intestine, which is quite an uncommon site [29]. Castronovo G et al. also reported the co-occurrence of GIST in HIV infection [30].

All the previously mentioned reports highlight the more elevated risk of malignant, aggressive and metastatic GIST in the case of HIV co-morbidity [28, 31]. Nevertheless, in our case, surgical removal of tumor was deferred due to the concomitant presence of multiple and severe OIs.

The reported case is unusual due to the number of opportunistic diseases (both infectious and tumoral) emerging in a short time span after the diagnosis of HIV infection. Actually, we counted as many as four opportunistic infections and one rare tumoral disease within two months from the first HIV diagnosis.

Late presenters HIV patients and AIDS presenters still represent a challenge for clinicians, which is often too complex to deal with and the risk of suboptimal results in spite of proper management should be taken into account. Prompt diagnosis followed by the immediate initiation of HAART certainly represents the best way to manage these patients.

## Additional files

Additional file 1: CARE checklist. (DOCX 1487 kb)

Additional file 2: case report clinical timeline. (DOCX 39 kb)
