# Supplementary material for: Rapid emergence of cryptococcal fungemia, Mycobacterium chelonae vertebral osteomyelitis and gastro intestinal stromal tumor in a young HIV late presenter: a case report
Source: BMC Infect Dis. 2018 Dec 27;18:693. doi: 10.1186/s12879-018-3573-z (PMC6307234; doi:10.1186/s12879-018-3573-z)
Supplement: Supplementary file 2 — case report clinical timeline. (DOCX 39 kb) [file 12879_2018_3573_MOESM2_ESM.docx]

March 24^th^

Interstitial pneumonia and ARDS

Transfer to ICU and death.

December 2017

Blood cultures resulted positive for Staph. Haemolyticus; started levofloxacin plus teicoplanin

Started liposomal amphothericin plus fluconazole

January 21^st^

Blood cultures resulted positive for Criptococcus neoformans; meningeal tests for Criptococcus were negative

January 20^th^

Total body CT scan showed bilateral lung nodular lesions with diffuse lymphadenopathy

January 19^th^

Transfer to our Unit.

Fever, intense back pain, low blood pressure, tachycardia, eupneic in room air, preserved neurocognitive functions.

Pancytopenia, high inflammatory marker levels

January 17^th^

Positive HIV test; High Viral Load; 5 T-CD4 cells/mm^3^

Start HAART

MRI showed vertebral lesions affecting multiple vertebral bodies

Admission in a peripheral hospital due to fever with chills associated with back pain in lumbar and dorsal area

January 30^th^

Oesophagogastroduodenoscopy with biopsy showed a gastric GIST

February 28^th^

PCR and culture on bone marrow and vertebral biopsy resulted positive for Non-tuberculous mycobacteria (M. chelonae). Started tobramycin plus clarithromycin.

February 19^th^

35-year-old caucasian male

Surgery for anal fissure in November 2017

Unremarkable clinical history
